# Disseminated* Mycobacterium interjectum* Infection with Bacteremia, Hepatic and Pulmonary Involvement Associated with a Long-Term Catheter Infection

**DOI:** 10.1155/2017/6958204

**Published:** 2017-01-18

**Authors:** David Sotello, D. Jane Hata, Mohammed Reza, Raj Satyanarayana, Vichaya Arunthari, Wendelyn Bosch

**Affiliations:** ^1^Division of Infectious Diseases, Mayo Clinic, Jacksonville, FL, USA; ^2^Department of Laboratory Medicine and Pathology, Mayo Clinic, Jacksonville, FL, USA; ^3^Department of Transplantation, Mayo Clinic, Jacksonville, FL, USA; ^4^Division of Pulmonary and Critical Care Medicine, Mayo Clinic, Jacksonville, FL, USA

## Abstract

We present a 49-year-old female with one year of intermittent fevers, chills, night sweats, and significant weight loss. Liver and lung biopsy showed evidence of a granulomatous process. Blood and liver biopsy cultures yielded growth of presumed* Mycobacterium interjectum*, thought to be related to a disseminated long-term central venous catheter infection. She successfully received one year of combined antimicrobial therapy after catheter removal without recurrence of disease.* M. interjectum* has been previously described as a cause of lymphadenitis in healthy children and associated with pulmonary disease in adults, although other localized infections have been reported. This is the first case described of a disseminated* M. interjectum* infection with bacteremia, hepatic and pulmonary involvement associated with a long-term catheter infection.

## 1. Introduction

Nontuberculous mycobacteria (NTM) are ubiquitous microorganisms that are an important cause of human disease, both in pediatric and in adult populations. These infections can occur in healthy or immunocompromised patients [1]. Advances in mycobacteriology techniques have allowed the identification of new NTM species [1], previously undiagnosed or misclassified [2, 3].* Mycobacterium interjectum* is an uncommon pathogen that has been associated with lymphadenitis in children and pulmonary involvement in adults [4, 5]. There are currently no specific guidelines for management of this uncommon infection. To our knowledge this is the first case report of disseminated* M. interjectum* infection.

## 2. Case

This is a 49-year-old female with past medical history of morbid obesity who underwent laparoscopic Roux-en-Y gastric bypass in 2005 and had a successful weight loss of approximately 68 kg. She had placement of a long-term central venous catheter in the right upper chest wall in 2010 for intermittent home hydration, poor venous access, and frequent laboratory monitoring by her local physicians. In 2012, she was hospitalized for acute interstitial pneumonia for which she required mechanical ventilation and systemic steroids; she eventually recovered from the respiratory failure. In 2013, she started to experience intermittent fevers (102-103°F) associated with occasional chills, night sweats, intermittent mild right upper quadrant pain, chronic nausea, vomiting, diarrhea, and involuntary weight loss of approximately 18 kg. Around the same time she was noted to have mildly elevated liver enzymes, which was previously attributed to fatty liver disease. Her liver enzymes continued to worsen which prompted further evaluation in 2013.

Physical examination revealed a temperature of 97.2°F, heart rate of 106 beats per minute, respiratory rate of 18 breaths per minute, blood pressure was 100/60 mmHg, height of 157 cm, and weight of 42.7 kg, and her body mass index was 17.3 kg/m^2^. She was not in acute distress, but appeared malnourished. A right chest wall tunneled long-term central venous catheter was in place without erythema, edema, or purulence around the exit site. She had mild hepatosplenomegaly, and the remainder of the examination was otherwise normal. The patient's initial pertinent laboratory workup results noted an alkaline phosphatase of 1,101 U/L (reference range 39–100 U/L), aspartate aminotransferase of 230 U/L (reference range 7–45 U/L), alanine aminotransferase of 118 U/L (reference range 8–43 U/L), angiotensin converting enzyme of 105 U/L (reference range 8–53 U/L), and lymphocytes flow cytometry with CD4 T cell count of 117 cells/*μ*L (reference range 424–1,509 cells/*μ*L). Antimitochondrial antibodies (AMA), antismooth muscle antibodies, immunoglobulin levels (IgG, IgA, and IgM), HIV 1/2 antibody, and viral hepatitis panel for types B and C were unremarkable. Computerized tomography (CT) of the chest showed mediastinal lymphadenopathy with bilateral mid and lower pulmonary infiltrates. A magnetic resonance elastography was done and showed hepatomegaly with changes consistent with stage 2 liver fibrosis. There were no strictures or beading to suggest sclerosing cholangitis and no focal lesions were noted.

**Figure 1:**
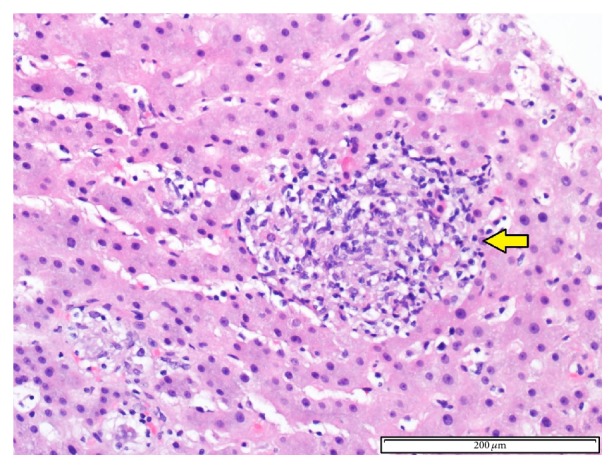
Hematoxylin and eosin staining (200x) of liver biopsy that demonstrates an epithelioid granuloma (arrow).

**Figure 2:**
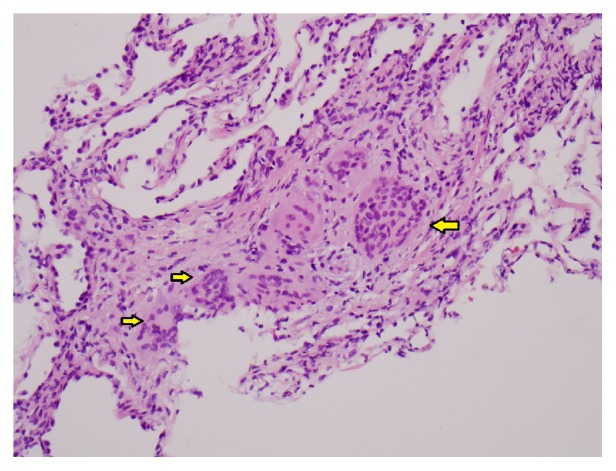
Hematoxylin and eosin staining (200x) of right lower lobe transbronchial biopsy showing nonnecrotizing granulomas (arrows).

Due to suspected AMA-negative primary biliary cirrhosis or infiltrative process like sarcoidosis, the patient underwent liver biopsy consistent with atypical sinusoidal T cell proliferation with scattered granulomas. The patient was evaluated by Oncology and underwent a bone marrow biopsy, which was negative for a lymphoproliferative disorder. The liver biopsy tissue specimen was cultured for acid-fast bacilli (AFB) which eventually yielded 2 colonies of a* Mycobacterium* species (sp.), not able to be identified by DNA sequencing. A second liver biopsy was notable for granulomatous hepatitis (Figure 1); however AFB tissue culture was negative. The patient was evaluated by Pulmonology and underwent transbronchial biopsy which showed rare nonnecrotizing granulomas (Figure 2), and AFB culture of bronchoalveolar lavage was negative. A transesophageal echocardiogram was negative for endocarditis.

AFB blood cultures obtained in 2013 from the long-term central venous catheter port and peripheral blood cultures yielded growth of* Mycobacterium* sp. Partial 16S ribosomal ribonucleic acid (rRNA) sequencing of the blood culture isolate indicated a 98% base pair (bp) match to* M. interjectum*. Sequencing of the* rpoB* gene indicated a 93.5% match (634/678 bp) to* M. interjectum*; according to Clinical and Laboratory Standards Institute (CLSI) guidelines for interpretation, this is only considered an identification to genus level. Sequencing of the* hsp65* gene was not performed. The long-term central venous catheter was removed and the catheter tip was sent for AFB culture, which also yielded growth of* Mycobacterium* sp.; this isolate was not sequenced.

Drug susceptibility testing was performed using a broth microdilution minimum inhibitory concentration (MIC) method as recommended by the CLSI [6]. From a pure patient isolate of* M. interjectum*, a suspension containing approximately 5 × 10^5^ CFU/mL was inoculated into cation-adjusted Mueller-Hinton broth containing serial dilutions of antimicrobial agent. Recommendations for standardized incubation conditions and times for slowly growing nontuberculous mycobacteria are not available. The MIC is reported as the lowest concentration of drug that inhibits visible growth, except in the case of trimethoprim/sulfamethoxazole, where 80% inhibition of growth is considered the end point.

The patient was initially treated with oral azithromycin 250 mg daily, ethambutol 800 mg daily, rifampin 450 mg daily, and intravenous amikacin 10 mg/kg/q24 h for 10 weeks. The patient then continued triple therapy with azithromycin, ethambutol, and rifampin for another 5 weeks. After* M. interjectum* susceptibilities were reported (Table 1), the therapy was modified to oral ciprofloxacin 500 mg twice a day, azithromycin 250 mg daily, and rifabutin 300 mg daily for additional 9 months. Moxifloxacin was not included in her treatment due to medication cost. AFB blood cultures obtained at 6 weeks and 3 months after removal of the long-term central venous catheter and initiation of antibiotic treatment were finalized as negative. Repeat CT of the chest four months after starting therapy showed resolution of pulmonary infiltrates and mediastinal lymphadenopathy. Her CD4 T cell count normalized and liver function tests returned to baseline. The patient was clinically monitored until 8 months after completion of therapy with repeat AFB blood cultures which were finalized as negative. At this point she had experienced a weight gain of 5 kg.

## 3. Discussion

*Mycobacterium interjectum* is a slow growing NTM, which owes its name to the phylogenetic position between rapid and slow growing mycobacteria [3]. Its colonies were originally described as smooth, scotochromogenic, and 1-2 mm in diameter [3]. The identification of mycobacteria was traditionally based upon phenotypic and biochemical characteristics, nucleic acid probes (available for only a limited number of NTM), and high-performance liquid chromatography [7], all of which have been largely supplanted by 16S rRNA sequencing [8].* M. interjectum* identification has been unsuccessful with biochemical and phenotypic techniques [7], since it has similar characteristics with other mycobacteria such as* M. scrofulaceum*,* M. gordonae*, and* M. simiae* [5, 7, 9]. Species level identification usually requires 16S rRNA gene sequencing [5, 7], although use of sequence data from the* hsp65* or* rpoB* genes has been described to be valuable tool in AFB identification [8, 10].

In our case, sequencing of the AFB blood culture isolate indicated a 98% 16S rRNA match to* M. interjectum* (449/458 bp). According to CLSI guidelines, sequence identity of 99% is suggested for genus-level identification, and 100% identity is required for species level identification. In our case a mismatch of 4 bp prevented identification of the isolate to genus level and resulted in a presumptive identification of* M. interjectum* based on sequence homology [11]. We did not perform PCR restriction fragment length polymorphism analysis for the* hsp65* gene which could have added a definite species level identification.

*Mycobacterium interjectum* rarely causes clinical disease in humans. It was originally first described in 1993 in an 18-month-old boy with lymphadenitis [3]. At least ten cases of* M. interjectum* lymphadenitis have been reported since then in very young immunocompetent children [4], possibly from sources such as oral exposure to contaminated objects [12].* M. interjectum* has also been isolated in sputum cultures of asymptomatic patients [7, 13] and from the gastrointestinal tract in a patient with acquired immunodeficiency syndrome [14]. In adults, infection with* M. interjectum* has been reported with pulmonary infection [5, 15, 16], cutaneous infection [17], and meningitis [18] (Table 1).

Our case is the first reported disseminated* M. interjectum* catheter related infection, presenting with mycobacteremia, granulomatous hepatitis, and pulmonary involvement in a malnourished patient. The patient's immunologic workup was negative for an immunodeficiency disorder. The initial abnormally low CD4 T cell count was likely related to her mycobacterial infection. Her liver function tests returned to baseline levels, her mycobacteremia resolved after the removal of central venous catheter, and she had resolution of pulmonary abnormalities noted on CT. Empiric antimicrobial agents were initially selected based on previous case reports. Once susceptibilities were available, targeted antimycobacterial therapy was modified accordingly to complete one year of treatment (Table 1).

There are currently no established guidelines for treatment of* M. interjectum* infection. Therapy may vary significantly depending on the host characteristics, location of infection, and susceptibility of the organism (Table 1). Infection in the pediatric population usually has occurred in otherwise healthy children, with most lymphadenitis cases requiring only surgical excision [4]. In reported adult cases of* M. interjectum* infection, treatment has been more complex due to the patient's age, medical comorbidities, immunocompromised states, and sites of infection, usually requiring prolonged combined antimicrobial regimen and/or surgical resection (Table 1). Fukuoka et al. successfully treated an isolated* M. interjectum* cutaneous infection with surgical intervention alone in a patient with microscopic polyangiitis on chronic steroid and azathioprine therapy [17]. Pulmonary and meningeal infections have previously been treated with prolonged courses of combined antibiotic therapy [5, 18].* M. interjectum* is known to be multidrug resistant [17]. Combination antibiotic therapy is usually required, with at least two or three active agents, which may include rifamycins, macrolides, and quinolones. Treatment duration has varied significantly amongst the known reported cases, usually between one and four years of therapy (Table 1).

To our knowledge this is the first reported case of disseminated* M. interjectum* catheter related infection, with mycobacteremia and granulomatous hepatic/pulmonary infection. Our patient was treated successfully with central venous catheter removal and one year of combination targeted antimicrobial therapy.

## Figures and Tables

**Table 1:** Reported *Mycobacterium interjectum* cases in adults.

Case	Age (years/sex)	Ref	Location	Symptoms	Predisposing conditions	Treatment	Outcome	Resistance pattern
(1)	74/F	[2]	Lungs	Persistent cough	History of possible tuberculosis and obstructive and restrictive pulmonary disease	R, E, and sulfamethoxazole for 4 years → inhaled Am indefinitely	Improvement of symptoms	Res: H, E, PAS, aminoglycosides, and quinolones Sus: Clr, Cfz, R, and Rfb
(2)	62/M	[5]	Lung	2 months of cough, night sweats, and weight loss	Tobacco use	Am, TMP/SMX, and R for 2 months → Clr, TMP/SMX, R, and E to complete 18 months	Cured	Sus: R, Rfb, Clr, S, Cfz, Am, and TMP/SMX Res: E, Cip
(3)	52/M	[15]	Lung	2 weeks of fever, malaise, and hemoptysis	Smoker	R, H, Z, and E for 2 months → R, H to complete one year	Cured	Res: R, E
(4)	48/M	[16]	Lung	2 weeks of cough, night sweats, and hemoptysis	None	R, H, and Z for 8 weeks → R, H for 4 months → after recurrence he was started on Clr, Lfx, R, and S for 9 months (planned for 2 years)	Recurrence at 18 months later. Eventually improved	Res: R, H, Z, E, Lzd, and Dox Sus: Clr, Lfx, and S
(5)	77/F	[17]	Skin	Subcutaneous nodules and abscesses	Microscopic polyangiitis, diabetes, chronic interstitial pneumonia, chronic steroids, and azathioprine	Surgical excision	Cured	Res: S, E, Km, H, R, Lfx, Clr, ethionamide, and Am
(6)	49/F	[18]	Cerebrospinal fluid	6 months of weight loss, visual disturbances, anorexia, poor memory, and episodic headaches	Alcoholism, coinfection with *Mycobacterium malmoense*	R, H, Z, and E for 2 years (Dexamethasone initially used)	Improved	N/A
(7)	49/F	Our case	Blood, liver, and catheter tip	1 year of fever/chills/night sweats and weight loss	Malnutrition secondary to bariatric surgery	Azm, E, R, and Am for 10 weeks → Azm, E, and R for 5 weeks → Azm, Cip, and Rfb for 9 months	Cured	Sus; Am, Cip, Clr, Lzd, Rfb, Mfx, and TMP/SMX Res: Dox, E, and R

Ref: reference, Res: resistant, Sus: susceptible, Am: amikacin, Azm: azithromycin, H: isoniazid, R: rifampin, Rfb: rifabutin, E: ethambutol, Clr: clarithromycin, Cip: ciprofloxacin, Cfz: clofazimine, Dox: doxycycline, Z: pyrazinamide, PAS: *p*-aminosalicylic acid, Lfx: levofloxacin, Lzd: linezolid, S: streptomycin, TMP/SMX: trimethoprim/sulfamethoxazole, →: followed by, N/A: not available.
